# A Novel Ranging and IMU-Based Method for Relative Positioning of Two-MAV Formation in GNSS-Denied Environments

**DOI:** 10.3390/s23094366

**Published:** 2023-04-28

**Authors:** Jia Cheng, Peng Ren, Tingxiang Deng

**Affiliations:** School of Telecommunications Engineering, Xidian University, Xi’an 710071, China; 21011210141@stu.xidian.edu.cn (J.C.); 21011210317@stu.xidian.edu.cn (T.D.)

**Keywords:** relative positioning, GNSS-denied environments, ranging, IMU, EKF, MAVs

## Abstract

Global Navigation Satellite Systems (GNSS) with weak anti-jamming capability are vulnerable to intentional or unintentional interference, resulting in difficulty providing continuous, reliable, and accurate positioning information in complex environments. Especially in GNSS-denied environments, relying solely on the onboard Inertial Measurement Unit (IMU) of the Micro Aerial Vehicles (MAVs) for positioning is not practical. In this paper, we propose a novel cooperative relative positioning method for MAVs in GNSS-denied scenarios. Specifically, the system model framework is first constructed, and then the Extended Kalman Filter (EKF) algorithm, which is introduced for its ability to handle nonlinear systems, is employed to fuse inter-vehicle ranging and onboard IMU information, achieving joint position estimation of the MAVs. The proposed method mainly addresses the problem of error accumulation in the IMU and exhibits high accuracy and robustness. Additionally, the method is capable of achieving relative positioning without requiring an accurate reference anchor. The system observability conditions are theoretically derived, which means the system positioning accuracy can be guaranteed when the system satisfies the observability conditions. The results further demonstrate the validity of the system observability conditions and investigate the impact of varying ranging errors on the positioning accuracy and stability. The proposed method achieves a positioning accuracy of approximately 0.55 m, which is about 3.89 times higher than that of an existing positioning method.

## 1. Introduction

Micro Aerial Vehicles (MAVs), which refer to small Unmanned Aerial Vehicles (UAVs), have gained popularity in aerial robotics due to their small size, agility, and versatility. It is possible for MAVs to perform a wide range of tasks, including surveillance, inspection, mapping, and environmental monitoring. However, achieving coordinated missions for MAV formations is a challenging task, as it requires precise and reliable navigation and positioning capabilities. Furthermore, such missions involve complex maneuvers and high synchronization between multiple MAVs, and any errors or inaccuracies in navigation or positioning can result in mission failure or even accidents. Therefore, developing robust and efficient navigation and positioning methods is crucial for enabling MAVs to perform coordinated missions effectively and safely.

Urban or forest environments are renowned for their complexity and heterogeneity, which pose significant challenges to wireless communication systems due to issues such as multipath propagation and shadowing [1]. In this context, Hermosilla et al. [2] proposed the use of street-based urban metrics descriptions to quantify various spatial patterns of urban types constructed at different periods, and provided eight different types of urban environments (including urban, residential, and industrial areas) based on their structural characteristics, such as building height distribution or vegetation coverage. These differences may impact wireless signal propagation in different ways. For example, historic urban areas with lower building heights and narrower streets may experience more severe multipath effects, while emerging industrial areas with taller buildings and wider streets may face more severe signal blockage issues. As a result, there are numerous challenges in receiving Global Navigation Satellite System (GNSS) signals in urban or forest environments due to the weak anti-interference ability of GNSS [3]. Despite some related studies [4,5,6] having reduced the error in GNSS-based positioning systems through multi-information fusion technology, intentional interference may render GNSS unusable in certain specialized fields, such as military applications. Therefore, achieving relative positioning of MAVs in GNSS-denied scenarios has become a potentially fruitful area of research.

The common methods for achieving relative positioning in GNSS-denied environments can be classified into the following three categories: visual positioning [7,8,9,10,11,12,13,14,15,16,17], inertial navigation positioning [18,19,20,21,22], and radio positioning [23,24]. The development status of each category of positioning technology is systematically discussed below, and the advantages and limitations of each technology are analyzed, providing good inspiration for the study of relative positioning methods for MAVs in GNSS-denied environments.

The visual localization methods can be broadly categorized into map-based localization [7,8,9,10,11] and map-free localization [12,13,14,15,16,17], depending on whether prior visual maps are utilized. (Note that visual refers to visual information, which is typically collected using sensors such as cameras or laser scanners. Visual maps can be understood as maps constructed based on visual information and used for visual localization and navigation.) In the map-based localization, a pre-constructed visual map was used to aid in localization, and the steps involved in map construction and updating, image retrieval, feature point extraction and matching, and precise localization have been studied extensively [7,8,9,10]. The main focus of [7] was the construction of 3D maps, while reference [8] addressed map quality issues by removing outliers and tracking lanes. Map matching problems were mainly addressed in [9,10,11]. In contrast, map-free visual localization methods do not rely on prior visual maps and instead estimate the pose of the object and surrounding environment. This category can be further divided into Visual Simultaneous Localization and Mapping (VSLAM) [12,13,14,15] and Structure from Motion (SFM) [16,17]. The VSLAM is designed for real-time processing, making it well-suited for applications such as robotics and autonomous vehicles, while SFM prioritizes accuracy and is more appropriate for offline processing applications such as digital reconstruction of scenes [16]. Specific techniques within these categories have also been put forward. For example, an efficient distributed particle filter (EDPF) was proposed in [12] to address the difficulty of sampling high-dimensional state spaces in range-only SLAM. Reference [13] proposed a weight-optimized particle filter-based algorithm for monocular visual SLAM, which aimed to improve the slow environmental interference repair speed of traditional filtering SLAM algorithms. Three-level parallel optimization was adopted in [14], including the direct method, feature-based method, and pose graph optimization. The method in [15] involves two stages: the first stage implements a local SLAM process based on filtering techniques, while the second stage utilizes optimization-based techniques for constructing and maintaining a consistent global map of the environment, which includes addressing the loop closure problem. To estimate the state of a MAV, the main filtering technique employed is the Extended Kalman Filter (EKF). In terms of optimization techniques, three methods are used: a local bundle adjustment technique to minimize the total reprojection error, the minimization of the Perspective-n-Point (PnP) problem, and graph optimization to correct the global map. In [16], 3D point clouds of close-range images were generated using SFM technology. Homologous features between different images were found to determine the shooting direction and position of each image. Finally, reference [17] used optimization to enhance the convergence rate of SFM by retrieving maximum internal similarity between images. The chosen images and query results were used to reconstruct a three-dimensional scene and estimate relative camera positions with SFM.

The inertial navigation positioning is an autonomous method that provides accurate short-term navigation and positioning without relying on external information or emitting radiation. However, the general positioning system is prone to an error accumulation due to the second-order integration operation [18,19,20,21,22]. Various studies have been conducted to address this issue. For instance, a 3D trajectory planning method based on Particle Swarm Optimization-A star (PSO-A*) algorithm was proposed in [18] to solve the yaw problem of intelligent aircraft. In [19], the nonlinear error model was considered, and internal measurement information was utilized to correct the nonlinear error of inertial navigation. The zero-velocity detection algorithm was adopted to compensate for the accumulated error of pedestrian inertial navigation [20,21]. Additionally, reference [22] employed Convolutional Neural Networks (CNN) to reduce the error of MEMS IMU sensors.

There are various wireless positioning methods based on radio technology, mainly including infrared positioning [23], Ultra Wide Band (UWB) positioning [24], and radio frequency identification (RFID) positioning [25]. In [23], the angle of an object equipped with a positioning device was measured using an infrared laser beam emitted by a lighthouse base station, and the position of the object was calculated. An indoor positioning system based on improved adaptive Kalman filter (IAKF) was proposed in [24] that calculated the distance between the tag and the base stations using the time it took for UWB signals to travel, and then applied a triangulation algorithm to acquire the position of the tag. Similar to [24], reference [25] differs mainly in the method of distance measurement, using RFID technology to calculate the distance. In addition, Ref. [25] mentions that RFID positioning tags can be classified into two types, active and passive, depending on their power requirements. Passive tags mainly involve backscattering communication, and therefore do not require direct power supply, but they have limited communication range compared to active tags. Therefore, RFID technology is mostly used for indoor product tracking. Furthermore, the design, installation, and maintenance of RF navigation systems can be expensive and complex, which may pose challenges for deploying them in certain applications, such as small unmanned aerial vehicles.

In addition, multi-technology fusion is an important means to improve the positioning performance. An important trend in VSLAM is to integrate visual sensor data with other sensor data [26,27]. A method was proposed in [26] to integrate Inertial Measurement Unit (IMU) and dynamic VSLAM, which avoided the static assumption of common SLAM algorithms and solved the problems of fast vehicle motion and insufficient light. This method exhibited higher robustness compared with pure visual dynamic SLAM systems. In [27], a SLAM autonomous positioning algorithm combining magnetometer, IMU, and monocular camera was proposed to address the initialization instability and drift problem in the visual-inertial SLAM (VI-SLAM) algorithm. In wireless positioning, UWB technology is a potentially productive area of research [28,29,30] in multi-information fusion positioning due to its advantages such as strong penetration ability, low power consumption, small impact of multipath effects, and high positioning accuracy [31,32,33]. In [28], UWB ranging was used in an indoor positioning scenario, and the position was jointly estimated by fusing the ranging and IMU information through cooperative positioning, which reduced the position drift of Inertial Navigation System (INS). A relative positioning method based on trilateration was proposed for the multi-mobile user mutual positioning scenario [29], where the UWB ranging and IMU information were fused and integrated into a probabilistic framework for cooperative positioning fault recovery. In [30], the pedestrian navigation was realized based on IMU and UWB ranging, and a zero-velocity detection algorithm and single anchor point reference were used. Due to the inherent error drift of IMU, relying solely on IMU for inertial navigation positioning is uncommon. Typically, the fusion of multi-sensor information is required to reduce the positioning errors [26,27,28,29,30,31,32,33,34,35].

Authors have two observations on the existing research on positioning methods.

- In the visual-based positioning research, the positioning accuracy is weakened in low-light environments and line-of-sight limitations lead to poor system robustness. These factors pose significant challenges for MAVs to efficiently perform collaborative tasks in diverse environments.
- In the research on IMU-based relative positioning, the multi-sensor fusion is mostly employed to reduce the inertial drift. Solely relying on IMU for dead reckoning cannot achieve long-term high-precision positioning [18,19,20,21,22]. When fusing with radio information, there are restrictions on MAV cooperative formation tasks, such as the existence of zero-velocity detection [31,33] and fixed reference anchor [33]. In addition, there is a situation where two MAVs cannot be positioned [32].

Motivated by the above facts, this paper investigates a relative positioning method of a two-MAV cooperative formation fusing inter-vehicle distance and IMU information in GNSS-denied environments, which does not require the use of other auxiliary devices such as odometers, except for the IMU and the devices used for ranging. The main contributions of this paper are summarized as follows:

1. A novel method for relative positioning in GNSS-denied scenarios is proposed based on ranging and IMU information. The method utilizes the EKF algorithm to jointly estimate the relative positions of MAVs, providing continuous, precise, and reliable information for the formation without being affected by the error accumulation problem of IMU. Additionally, the method is capable of achieving relative positioning for an arbitrary number of nodes without requiring an accurate reference anchor. This innovative approach offers notable advantages over existing methods and has great potential for applications in various fields of aerial robotics.
2. Theoretical derivations of the system observability conditions are presented, along with the specific expressions. The conditions indicate that the system positioning accuracy and reliability can be guaranteed when the flight trajectory of the MAV formation satisfies the observability conditions. Failure to satisfy these conditions may result in decreased positioning accuracy and reliability, as well as a possibility of divergence.
3. Monte Carlo simulations were conducted, where the correctness of the system observability conditions was verified and the effects of different ranging errors on the positioning accuracy and reliability were investigated. Moreover, the positioning error was reduced by approximately 3.89 times compared to an existing positioning method [32].

The rest of this paper is organized as follows. Section 2 describes the system model. Section 3 presents a detailed description of the relative positioning method. Section 4 de-rives the system observability conditions and provides the specific expressions. Section 5 presents simulation results, followed by Section 6 which concludes this paper.

## 2. System Model

**Figure 1:**
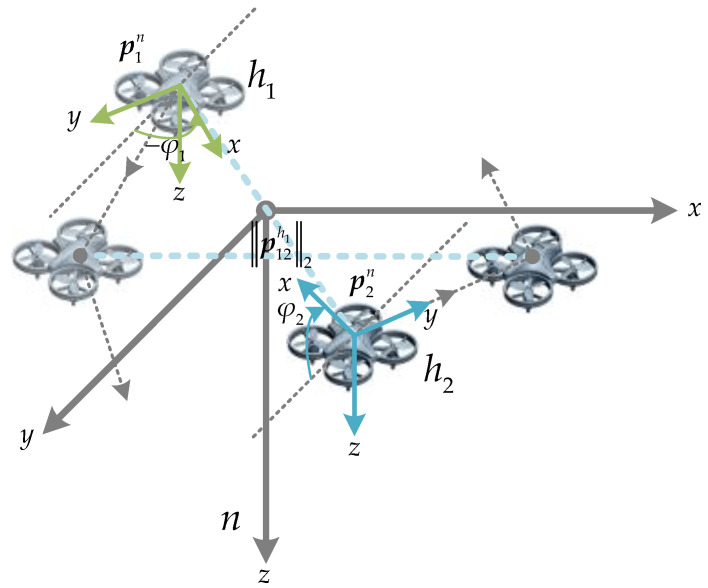
Description of the system model (Gray: NED coordinate system (*n*); Olive: body-fixed horizontal coordinate system ($h_{1}$); Light blue: body-fixed horizontal coordinate system ($h_{2}$ )).

In this paper, we consider a system model for relative localization of two cooperative MAVs in GNSS-denied environments, as shown in Figure 1. The definitions of the reference coordinate systems are explained as follows. The global coordinate system is the Earth-fixed North-East-Down (NED) coordinate system, denoted as *n*, and assumed to be an inertial frame. The origin of the body-fixed horizontal coordinate system (denoted as $h_{i}$, *i* = 1, 2) for MAV *i* (*i* = 1, 2) is located at its center of gravity, with *x*-*y* plane and *z*-axis paralleling those of *n*, respectively, meaning that $h_{i}$ is obtained by rotating the *n* around its *z*-axis by a yaw angle φ.

It is worth noting that this paper does not use the typical body-fixed coordinate system (denoted as $b_{i}$, *i* = 1, 2) for the MAV *i*, which is represented by Euler angles with respect to *n*. According to the 321-rotation sequence, the corresponding Euler angles are yaw angle φ, pitch angle θ, and roll angle γ, respectively. The reason for using $h_{i}$ instead of $b_{i}$ is to simplify the kinematic relationships and minimize the impact of unnecessary factors, such as near-hovering states with small roll and pitch angles.

In the system model, MAV *i* can measure its own state variables, which include acceleration, angular velocity, and velocity in $h_{i}$. Furthermore, by means of wireless communication, MAV *i* can also obtain velocity and distance information from the other one in the system framework, where two MAVs utilize inter-vehicle distance information to enhance the accuracy and robustness of relative positioning in the system framework.

The relative motion of two MAVs is described in *h*. Let $p_{i}^{n}$ denote the position vector of MAV *i* in *n*, where the subscript *i* refers to the MAV number and the superscript *n* indicates the vector is projected onto *n*. The projection of the relative position of MAV *k* (*k* = 1, 2, but k≠i) with respect to MAV *i* in $h_{i}$ can be expressed as:

(1) $$p_{ik}^{h_{i}}=C_{n}^{h_{i}}\left(p_{k}^{n}-p_{i}^{n}\right)$$

where $C_{n}^{h_{i}}$ is the coordinate transformation matrix from *n* to $h_{i}$ (see Equation (2)). It is only dependent on the yaw angle $\varphi_{i}$ of MAV *i*, since *x*-*y* plane and *z*-axis of $h_{i}$ are parallel to those of *n*, respectively.

(2) $$C_{n}^{h_{i}}=\left[\begin{array}{ccc} \cos\varphi_{i} & -\sin\varphi_{i} & 0\\ \sin\varphi_{i} & \cos\varphi_{i} & 0\\ 0 & 0 & 1 \end{array}\right]$$

It can be inferred from Equations (1) and (2) that the *z*-component of the relative position $p_{ik}^{h_{i}}$ corresponds to the difference in height (which can be measured by a barometric altimeter) between MAV *i* and MAV *k* in *n*. Therefore, the system model can be simplified from three-dimensional space to a two-dimensional plane in the horizontal direction.

Some of the parameters and symbols used in this section are shown in Table 1.

## 3. A Ranging and IMU-Based Relative Positioning Method

We propose a relative positioning method that integrates the inter-vehicle distance with on-board IMU information and employs the EKF algorithm for optimal position estimation. The acquired data is transformed into the corresponding framework to establish the state differential equation. Based on the measurement information, the state of the MAVs is updated. The system observability analysis is conducted before and after the EKF algorithm’s prediction and update process to ensure the system positioning accuracy. Specifically, in Section 3.1, the state differential equation between two MAVs is simply derived, and the observation model and the overall system process are given in Section 3.2 and Section 3.3, respectively.

### 3.1. State Differential Model

The nonlinear state vector system for the localization model is defined as:

(3) $$\left\{\begin{array}{l} \dot{x}=f\left(x,u\right)\\ y=h\left(x\right) \end{array}\right.$$

where the vectors $x\in {\mathbb{R}}^{n}$, $u\in {\mathbb{R}}^{m}$, $y\in {\mathbb{R}}^{l}$ are the state vector, input vector, and output vector of the system, respectively. $f\left(x,u\right)$ is the system state differential vector function containing the vector parameters ***x*** and ***u***, and $h\left(x\right)$ is the observation equation related to the state vector ***x***.

According to the discussion in Section 2, and considering the relative positioning model of two MAVs, let $p=R\left(p_{2}-p_{1}\right)\in {\mathbb{R}}^{2}$, which represents the projection of the relative position of MAV 2 with respect to MAV 1 in $h_{1}$, where ***R*** is the two-dimensional case of Equation (2). Specifically, it is equal to:

(4) $$R=\left[\begin{array}{cc} \cos\left(\Delta \varphi \right) & -\sin\left(\Delta \varphi \right)\\ \sin\left(\Delta \varphi \right) & \cos\left(\Delta \varphi \right) \end{array}\right]$$

where $\Delta \varphi =\varphi_{2}-\varphi_{1}$ is the yaw angle difference between MAV 1 and MAV 2.

Moreover, the other variables in the nonlinear system are defined below. The yaw rate difference is denoted by $\Delta \dot{\varphi }=r_{2}-r_{1}$ (where $r_{i}$ is the yaw rate of MAV *i*, *i* = 1, 2). The projections of velocity and acceleration of MAV *i* in $h_{i}$ are denoted as $v_{i}\in {\mathbb{R}}^{2}$ and $a_{i}\in {\mathbb{R}}^{2}$, respectively.

It should be noted that the yaw rate $r_{i}$ and acceleration $a_{i}$ mentioned earlier refer to the horizontal plane components in $h_{i}$ and cannot be equated simply with the actual measured values $\omega_{i}\in {\mathbb{R}}^{3}$ and $s_{i}\in {\mathbb{R}}^{3}$ of the gyroscope and accelerometer. In particular, the latter must undergo a conversion process, which can be expressed as:

(5) $$r_{i}=\frac{\sin\gamma_{i}}{\cos\theta_{i}}\cdot \omega_{iy}+\frac{\cos\gamma_{i}}{\cos\theta_{i}}\cdot \omega_{iz},\;i=1,\;2$$

(6) $$a_{i}=\left[\begin{array}{ccc} \cos\theta_{i} & \sin\gamma_{i}\sin\theta_{i} & \cos\gamma_{i}\sin\theta_{i}\\ 0 & \cos\gamma_{i} & -\sin\gamma_{i} \end{array}\right]\cdot s_{i},\;i=1,\;2$$

where $\omega_{iy}$ and $\omega_{iz}$ represent the *y*-axis component (true pitch rate) and *z*-axis component (true yaw rate) of $\omega_{i}$, respectively. $\gamma_{i}$ and $\theta_{i}$ are the roll angle and pitch angle of MAV *i*, respectively. Proofs of Equations (5) and (6) are provided in Appendix A for reference.

Let the state and input vector be $x={\left[p^{T},\Delta \varphi ,v_{1}^{T},v_{2}^{T}\right]}^{T}\in {\mathbb{R}}^{7}$ and $u={\left[r_{1},r_{2},a_{1}^{T},a_{2}^{T}\right]}^{T}\in {\mathbb{R}}^{6}$, respectively. The state differential equation of the system is simply derived as follows.

The derivative of the relative position ***p*** with respect to time *t* is (Note that in Equation (7), the coordinate transformation matrix ***R*** does not act on $v_{1}$. The reason is that, in the scenario of mutual positioning between two MAVs, when calculating the relative position of MAV 2 with respect to MAV 1 in $h_{1}$, there is no need to transform the velocity of MAV 1 in its own coordinate system $h_{1}$ by left-multiplying ***R***):

(7) $$\begin{array}{ll} \frac{dp}{dt} & =\frac{dR\left(p_{2}-p_{1}\right)}{dt}\\ & =\frac{dR}{dt}\left(p_{2}-p_{1}\right)+R\frac{d\left(p_{2}-p_{1}\right)}{dt}\\ & =-r_{i}^{\times }R\left(p_{2}-p_{1}\right)+Rv_{2}-v_{1}\\ & =-r_{1}^{\times }p+Rv_{2}-v_{1} \end{array}$$

where the antisymmetric matrix $r_{i}^{\times }$ of cross product in two-dimensional case is equal to:

(8) $$r_{i}^{\times }=\left[\begin{array}{cc} 0 & -r_{i}\\ r_{i} & 0 \end{array}\right],\;i=1,\;2$$

$v_{i}$ is obtained by coordinate transformation of $v_{i}^{n}\in {\mathbb{R}}^{2}$ in *n*, which is equal to $v_{i}=Rv_{i}^{n}$. Taking the derivative of the velocity $v_{i}$ with respect to time *t*:

(9) $$\begin{array}{cc} \frac{dv_{i}}{dt} & =\frac{dRv_{i}^{n}}{dt}\\ & =\frac{dR}{dt}v_{i}^{n}+R\frac{dv_{i}^{n}}{dt}\\ & =-r_{i}^{\times }Rv_{i}^{n}+a_{i}\\ & =-r_{i}^{\times }v_{i}+a_{i} \end{array}$$

According to Equations (7) and (9), and the definition of the yaw rate difference $\Delta \dot{\varphi }$, the state differential equation in Equation (3) can be written as:

(10) $$\dot{x}=f\left(x,u\right)=\left[\begin{array}{c} -r_{1}^{\times }p+Rv_{2}-v_{1}\\ r_{2}-r_{1}\\ -r_{1}^{\times }v_{1}+a_{1}\\ -r_{2}^{\times }v_{2}+a_{2} \end{array}\right]\in {\mathbb{R}}^{7}$$

### 3.2. Observation Model

The observations involved in the positioning model are the distance information ${\left\|p\right\|}_{2}$ between two MAVs and the velocity $v_{i}$ of MAV *i* (where *i* = 1, 2). Converting the distance information to ${\left\|p\right\|}_{2}^{2}/2$ can simplify the system observability theoretical analysis in Section 4 without affecting the analysis results [36]. However, the distance observation is still selected as ${\left\|p\right\|}_{2}$ in the experimental verification. The corresponding observation model is expressed as:

(11) $$y=h\left(x\right)=\left[\begin{array}{c} {\left\|p\right\|}_{2}^{2}/2\\ v_{1}\\ v_{2} \end{array}\right]\in {\mathbb{R}}^{5}$$

### 3.3. Overall System Process

**Figure 2:**
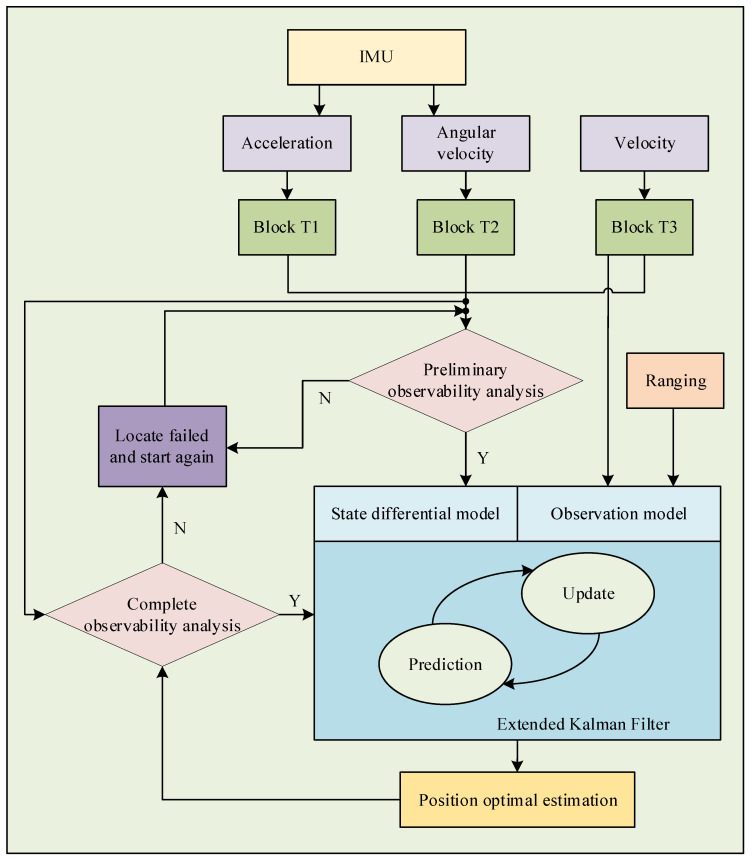
The overall system process (including information acquisition and transformation, preliminary observability analysis, EKF algorithm, and complete system observability analysis).

The system process as a whole is depicted in Figure 2. In the system framework illustrated in Figure 1, the MAVs acquire their motion states through onboard sensors, encompassing acceleration, angular velocity, and velocity, which are transformed into $h_{1}$ via transformation Blocks T1, T2, and T3, respectively.

Subsequently, a preliminary observability analysis is conducted to determine whether the observability conditions presented in Equations (30)–(32) of Section 4 are satisfied. Provided that the conditions are met, the positioning accuracy is adequately guaranteed. Otherwise, the system fails to localize and awaits the next sampling interval to retry localization.

One important aspect of the system process is that the state differential equation is constructed based on the MAVs’ states. In addition, the real-time distance measurement between two MAVs and the target MAV’s velocity are obtained through radio communication, with the measurement equation constructed accordingly. The EKF algorithm is then used to estimate the optimal relative position of the target MAV.

A complete system observability analysis is performed based on the optimal position estimation and the MAVs’ states, as shown in Equation (29). The result is expected to be similar to that of the preliminary observability analysis, with the only difference being the expression of the conditions.

Some of the parameters and symbols used in this section are shown in Table 2.

## 4. System Observability Analysis

Considering the nonlinear state vector system shown in Equation (3), the system observability is analyzed by means of Lie derivative. The multiple Lie derivatives are defined as follows:

(12) $$L_{f}^{0}h=h$$

(13) $$L_{f}^{i}h=L_{f}\left(L_{f}^{i-1}h\right)=J\left(L_{f}^{i-1}h\right)\cdot f,\;i\in {\mathbb{N}}^{*}$$

where $J\left(L_{f}^{i}h\right)$ represents the Jacobi matrix of $L_{f}^{i}h$. Furthermore, the observability matrix of the nonlinear system is:

(14) $$H=\left[\begin{array}{c} J\left(L_{f}^{0}h\right)\\ J\left(L_{f}^{1}h\right)\\ \vdots \\ J\left(L_{f}^{i}h\right) \end{array}\right],\;i\in {\mathbb{N}}^{*}$$

When the observable matrix ***H*** has full rank, the nonlinear system is considered locally weakly observable [37].

The first term of the observability matrix ***H*** is equal to:

(15) $$J\left(L_{f}^{0}h\right)=J\left(h\right)=\left[\begin{array}{cccc} p^{T} & 0 & 0_{1\times 2} & 0_{1\times 2}\\ 0_{2\times 2} & 0_{2\times 1} & E_{2} & 0_{2\times 2}\\ 0_{2\times 2} & 0_{2\times 1} & 0_{2\times 2} & E_{2} \end{array}\right]=\left[\begin{array}{ccc} p^{T} & 0 & 0_{1\times 4}\\ 0_{4\times 2} & 0_{4\times 1} & E_{4} \end{array}\right]$$

Since the cross term of the last four rows and columns in the first term of the observability matrix ***H*** is the unit matrix, the rank of ***H*** cannot be increased by its corresponding observation functions. Therefore, only the distance observation corresponding to the first row in Equation (11), denoted as $h_{1}\left(x\right)={\left\|p\right\|}_{2}^{2}/2$, needs to be considered.

The first-order Lie derivative corresponding to the distance observation $h_{1}\left(x\right)$ is equal to:

(16) $$L_{f}^{1}h_{1}=J\left(L_{f}^{0}h_{1}\right)\cdot f=p^{T}\left(-r_{1}^{\times }p+Rv_{2}-v_{1}\right)$$

Calculating the Jacobi matrix of the first-order Lie derivative, the second term of the observability matrix ***H*** can be obtained:

(17) $$J\left(L_{f}^{1}h_{1}\right)={\left[\begin{array}{c} \left(Rv_{2}-v_{1}\right)\\ p^{T}\frac{\partial R}{\partial \Delta \varphi }v_{2}\\ -p\\ R^{T}p \end{array}\right]}^{T}$$

Given that the full rank of the observability matrix ***H*** is seven and the first term $J\left(L_{f}^{0}h\right)$ has rank five, calculation of the second-order Lie derivative is necessary. The second-order Lie derivative is given by:

(18) $$\begin{array}{ll} L_{f}^{2}h_{1} & =J\left(L_{f}^{1}h_{1}\right)\cdot f\\ & ={\left(Rv_{2}-v_{1}\right)}^{T}\left(-r_{1}^{\times }p+Rv_{2}-v_{1}\right)+p^{T}\frac{\partial R}{\partial \Delta \varphi }v_{2}\left(r_{2}-r_{1}\right)\\ & \;-p^{T}\left(-r_{1}^{\times }v_{1}+a_{1}\right)+p^{T}R\left(-r_{2}^{\times }v_{2}+a_{2}\right)\\ & =v_{2}^{T}v_{2}-2v_{1}^{T}Rv_{2}+v_{1}^{T}v_{1}+p^{T}\frac{\partial R}{\partial \Delta \varphi }v_{2}r_{2}-p^{T}a_{1}+p^{T}R\left(-r_{2}^{\times }v_{2}+a_{2}\right) \end{array}$$

where the simplification is achieved using Equations (19) and (20). The result shows that the yaw rate $r_{1}$ of MAV 1 is completely cancelled out.

(19) $$R^{T}R=E_{2}$$

(20) $$R^{T}r_{1}^{\times }=-\frac{\partial R^{T}}{\partial \Delta \varphi }r_{1}$$

The third term of the observability matrix ***H*** is the Jacobi matrix of the second-order Lie derivative (see Equation (18)), which can be expressed as:

(21) $$J\left(L_{f}^{2}h_{1}\right)=\left[\begin{array}{cccc} \frac{\partial L_{f}^{2}h_{1}}{\partial p} & \frac{\partial L_{f}^{2}h_{1}}{\partial \Delta \varphi } & \frac{\partial L_{f}^{2}h_{1}}{\partial v_{1}} & \frac{\partial L_{f}^{2}h_{1}}{\partial v_{2}} \end{array}\right]$$

where the specific expressions of each term are derived simply as follows:

(22) $$\begin{array}{ll} \frac{\partial L_{f}^{2}h_{1}}{\partial p} & =v_{2}^{T}\left(\frac{\partial R^{T}}{\partial \Delta \varphi }r_{2}-r_{2}^{\times T}R^{T}\right)-a_{1}^{T}+a_{2}^{T}R^{T}\\ & =-a_{1}^{T}+a_{2}^{T}R^{T} \end{array}$$

(23) $$\begin{array}{ll} \frac{\partial L_{f}^{2}h_{1}}{\partial \Delta \varphi } & =-2v_{2}^{T}\frac{\partial R^{T}}{\partial \Delta \varphi }v_{1}+v_{2}^{T}\left(\frac{\partial^{2}R^{T}}{\partial \Delta \varphi^{2}}r_{2}-r_{2}^{\times T}\frac{\partial R^{T}}{\partial \Delta \varphi }\right)p+a_{2}^{T}\frac{\partial R^{T}}{\partial \Delta \varphi }p\\ & =-2v_{2}^{T}\frac{\partial R^{T}}{\partial \Delta \varphi }v_{1}+a_{2}^{T}\frac{\partial R^{T}}{\partial \Delta \varphi }p \end{array}$$

(24) $$\frac{\partial L_{f}^{2}h_{1}}{\partial v_{1}}=-2v_{2}^{T}R^{T}+2v_{1}^{T}$$

(25) $$\begin{array}{ll} \frac{\partial L_{f}^{2}h_{1}}{\partial v_{2}} & =2v_{2}^{T}-2v_{1}^{T}R+p^{T}\left(\frac{\partial R}{\partial \Delta \varphi }r_{2}-Rr_{2}^{\times }\right)\\ & =2v_{2}^{T}-2v_{1}^{T}R \end{array}$$

It can be seen from the above simplified results that the yaw rate $r_{2}$ of MAV 2 is completely offset. In the event that the combination matrix ***A*** consisting of the Jacobi matrixes of the zero-order, first-order, and second-order Lie derivatives corresponding to the distance observation $h_{1}\left(x\right)$ has full rank, the rank of observability matrix ***H*** is guaranteed to have full rank, indicating that the nonlinear system is observable.

(26) $$A=\left[\begin{array}{cc} p^{T} & 0\\ v_{2}^{T}R^{T}-v_{1}^{T} & p^{T}\frac{\partial R}{\partial \Delta \varphi }v_{2}\\ -a_{1}^{T}+a_{2}^{T}R^{T} & -2v_{1}^{T}\frac{\partial R}{\partial \Delta \varphi }v_{2}+p^{T}\frac{\partial R}{\partial \Delta \varphi }a_{2} \end{array}\right]\in {\mathbb{R}}^{3\times 3}$$

In light of the above discussion, the observable system needs to satisfy the following condition:

(27) $$\left|A\right|\neq 0$$

Multiplying each element in the third column of determinant $\left|A\right|$ with the corresponding algebraic cofactor, adding and expanding to calculate, we can obtain the following expression:

(28) $$\begin{array}{ll} \left|A\right| & =-p^{T}\frac{\partial R}{\partial \Delta \varphi }v_{2}\cdot \left(-a_{1}^{T}+a_{2}^{T}R^{T}\right)R_{\Delta \varphi =\pi /2}p\\ & \;+\left(-2v_{1}^{T}\frac{\partial R}{\partial \Delta \varphi }v_{2}+p^{T}\frac{\partial R}{\partial \Delta \varphi }a_{2}\right)\cdot \left(v_{2}^{T}R^{T}-v_{1}^{T}\right)R_{\Delta \varphi =\pi /2}p\\ & =\left(p^{T}\frac{\partial R}{\partial \Delta \varphi }\left(v_{2}a_{1}^{T}-a_{2}v_{1}^{T}\right)-2v_{1}^{T}\frac{\partial R}{\partial \Delta \varphi }\left(v_{2}v_{2}^{T}R^{T}-v_{2}v_{1}^{T}\right)\right)R_{\Delta \varphi =\pi /2}p \end{array}$$

Considering the properties of matrix $R_{\Delta \varphi =\pi /2}$, when p≠0 and Equation (28) satisfies the following inequality (where *m* is an arbitrary constant), Equation (27) holds, and the nonlinear system is observable.

(29) $$p^{T}\frac{\partial R}{\partial \Delta \varphi }\left(v_{2}a_{1}^{T}-a_{2}v_{1}^{T}\right)-2v_{1}^{T}\frac{\partial R}{\partial \Delta \varphi }\left(v_{2}v_{2}^{T}R^{T}-v_{2}v_{1}^{T}\right)\neq mp^{T}$$

While the inequality (29) is not intuitive in revealing the motion constraints of MAVs, it can be used to extract some more obvious conditions (as seen in Equations (30)–(32)), which greatly aid our comprehension of Equation (29).

(30) p≠0

(31) $$v_{i}\neq 0\;or\;a_{i}\neq 0,\;i=1,\;2$$

(32) $$v_{1}\neq nRv_{2}\;or\;\left(a_{1}\neq 0\;or\;a_{2}\neq 0\right)$$

Equation (30) serves as a prerequisite for Equation (29), which means that the relative position between two MAVs cannot be equal to zero. Equation (31) indicates that the MAVs cannot remain stationary. Both the conditions are obvious. Equation (32) shows that two MAVs cannot fly in parallel unless at least one of the MAVs has a non-zero acceleration, where *n* is an arbitrary constant.

According to the system observability analysis in this section, we derived the motion conditions that must be satisfied by MAVs (see Equations (29)–(32)), which means if the relative motion between two MAVs in the system violates the observability conditions, the positioning accuracy and reliability of MAVs cannot be guaranteed. In such a case, by actively intervening in the relative motion of MAVs to satisfy the observability conditions, MAVs in the system can still achieve mutual positioning.

Some of the parameters and symbols used in this section are shown in Table 3.

## 5. Results

In this section, we employ computer simulations to demonstrate the performance of the proposed relative positioning method. Section 5 is structured as follows: Section 5.1 describes the experimental setup, followed by Section 5.2 which verifies the system observability conditions. In Section 5.3, we investigate the impact of different ranging errors on the positioning accuracy and stability. Finally, an error comparison experiment with an existing method is conducted in Section 5.4.

### 5.1. Experimental Setup

Assuming two MAVs, *A* and *B*, in the system framework depicted in Figure 1, we use the proposed relative positioning method to calculate the position of MAV *B* relative to MAV *A*. The specific parameter settings for the simulation experiment are listed in Table 4, where deg=π/180 and $mg=9.8\times 10^{-3}$.

Explanations for certain simulation parameters in Table 4 are provided below. The gyroscope and accelerometer parameters are typical electrical parameters of commercial IMU inertial sensors (such as MPU6050) commonly available on the market [38]. The error of the barometric altimeter was referenced from [39], where an ultrahigh resolution pressure sensor based on percolative metal nanoparticle arrays was designed. The sensor has an ultra-high resolution of 0.5 Pa and high sensitivity of $0.13\;{\mathrm{kPa}}^{-1}$, and the pressure range and sensitivity can be adjusted by changing the thickness of the PET membrane, which extends the working pressure range to 40 kPa. In actual altitude tests, the altitude measurement sensitivity was calculated as $-0.00025\;m^{-1}$ according to the slope of the response curve. RMS noise analysis shows that the accuracy of the sensor can be as low as 1 m. As a high-precision barometric sensor, the sensor can be used for high-resolution barometric altimeters (Note that it is possible to have different pressure at the same height but at different horizontal locations. However, in this work, we mainly focus on the relative positioning of two MAVs, where two MAVs are generally not distant from each other. Thus, it is reasonable to assume that the pressure difference of two MAVs only comes from the altitude level) [39]. The ranging error in this paper is referenced from [40], which proposed an improved through-the-wall (TTW) NLOS ranging method using UWB technology to achieve ranging errors of 0–2 m in NLOS environments. In addition, the noise used in the simulation is close to actual noise, and an adaptive adjustment method is employed for the noise matrix of the system to better adapt to the changes in noise characteristics and maintain good filtering performance.

In accordance with the system observability analysis presented in Section 4, the flight trajectory of the two MAVs needs to satisfy Equation (29) to guarantee the system positioning accuracy and reliability. A trajectory satisfying the requirements of Equation (29), called Trajectory 1, can be defined as follows: the straight-line *AB* rotates around the geometric center *O* in the horizontal plane, while *O* undergoes sinusoidal motion. The MAV height is measured using a barometric altimeter. The motion parameters are listed in Table 5. Setting the rotational angular velocity of *AB* and sinusoidal angular velocity of *O* to zero in Table 5, results in a straight-line flight of the MAVs in parallel, which is denoted as Trajectory 2.

### 5.2. Verification of the System Observability Conditions

Section 5.2 aims to validate the system observability conditions derived in Section 4 and evaluate the effectiveness of the proposed relative positioning method.

In Section 5.1 of the experimental setup, two trajectories for the MAVs are defined: Trajectory 1 and Trajectory 2. Trajectory 1 satisfies the system observability condition shown in Equation (29), while Trajectory 2 fails to comply with it. More specifically, Trajectory 2 violates the observability sub-condition shown in Equation (32).

**Figure 3:**
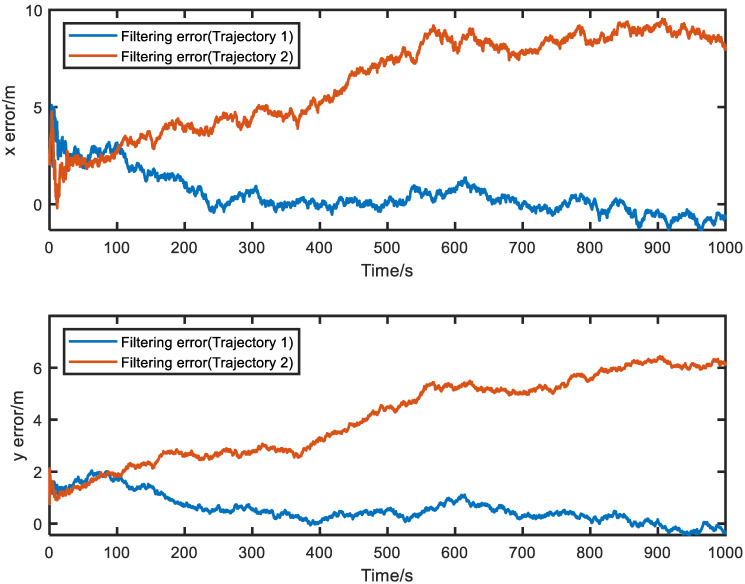
Filtering errors for the two trajectories.

**Figure 4:**
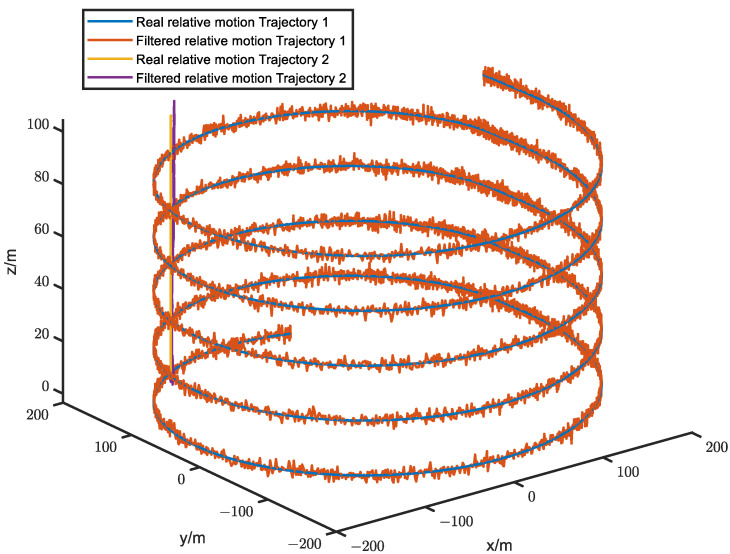
The relative motion trajectories of MAV *B* relative to *A*.

Figure 3 shows the filtered error results for the two trajectories, while the corresponding relative motion trajectories are presented in Figure 4. The average filtering errors for *x* and *y* axes of Trajectory 1 are approximately 1.22 m and 0.57 m, respectively, with the errors gradually converging to zero over time. In contrast, Trajectory 2 has significantly larger average filtering errors of 6.23 m and 3.86 m for *x* and *y* axes, respectively, exceeding those of Trajectory 1 by factors of 5.1 and 6.77. Furthermore, the errors of Trajectory 2 tend to diverge over time. These results indicate that system observability conditions are critical for ensuring the higher positioning accuracy and reliability, whereas failing to meet it, the system accuracy and reliability cannot be guaranteed.

**Figure 5:**
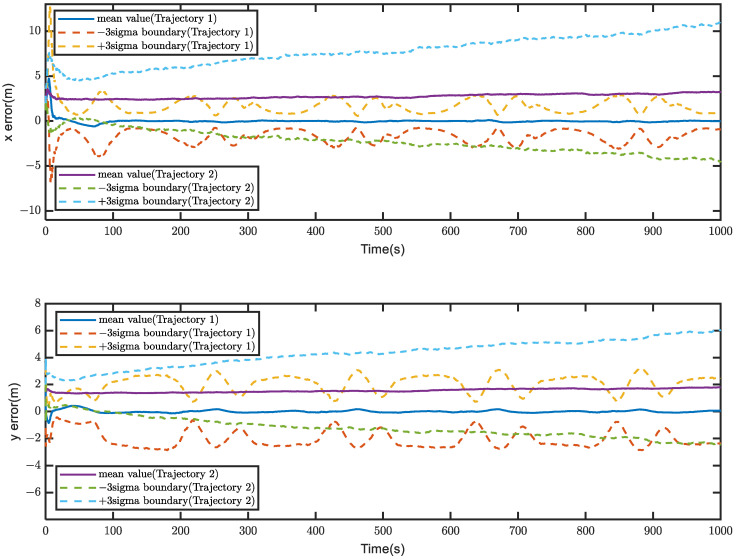
100 Monte Carlo simulations, which are conducted to verify the system observability conditions.

For the purpose of avoiding experimental randomness, Monte Carlo simulation experiments were conducted to investigate the error statistical characteristics of Trajectory 1 and Trajectory 2. Figure 5 presents the results of 100 Monte Carlo simulation experiments for error statistics. The upper and lower dashed lines of the error curves represent the +3σ and −3σ boundaries, respectively, while the solid lines represent the error mean. The boundary values represent the positioning accuracy, and the boundary range represents the positioning stability. The specific data for Figure 5 is listed in Table 6. It can be concluded from Figure 5 and Table 6 that the positioning accuracy and stability of Trajectory 2 are worse than those of Trajectory 1, and the positioning error of Trajectory 2 exhibits a diverging trend.

The simulation experiments in Section 5.2 demonstrate that the system has smaller positioning errors when it satisfies the observability conditions, versus larger errors with a divergent trend when it fails to meet the conditions. The result further confirms the validity of the system observability theoretical analysis in Section 4.

### 5.3. The Influence of Different Ranging Errors on the Positioning Accuracy and Stability

**Figure 6:**
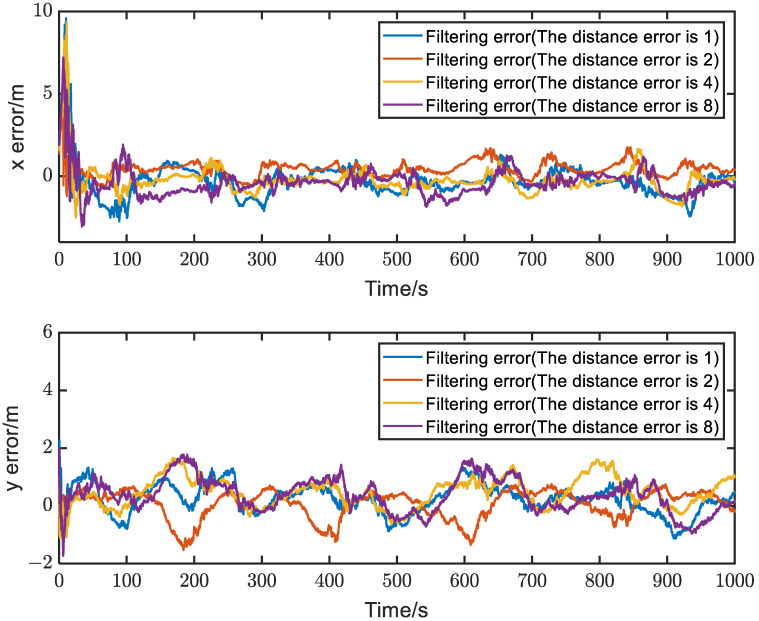
The positioning errors corresponding to different ranging errors, where four ranging error levels of 1 m, 2 m, 4 m, and 8 m are set.

In this section, we investigate the impact of different ranging errors on the positioning accuracy and stability of the proposed relative localization method, which integrates ranging and IMU information. The ranging errors for Trajectory 1 are set to four levels: 1 m, 2 m, 4 m, and 8 m. The results of a single experiment’s positioning errors are shown in Figure 6. Since Trajectory 1 satisfies the system observability conditions, the corresponding positioning accuracy is within 2 m after approximately 100 s, despite the different ranging errors, and the system remains stable.

**Figure 7:**
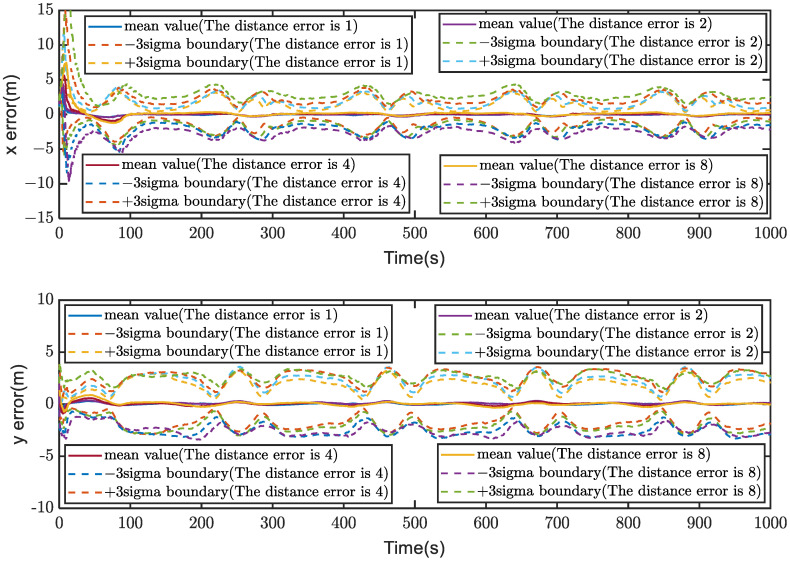
100 Monte Carlo simulations, which are conducted to investigate the impact of different ranging errors on the positioning accuracy and stability.

Subsequently, 100 Monte Carlo simulations are conducted, and the simulation results are presented in Figure 7. It can be visually observed that the larger the ranging error, the worse the system’s positioning accuracy and stability. However, increasing ranging errors did not cause the positioning accuracy and stability of the proposed system to deteriorate significantly, meaning the system did not diverge, but only increased the error. Specifically, the average boundary value increased from about 2 m to around 3 m as ranging errors increased from 1 m to 8 m. These experimental results indicate that different ranging errors have a limited impact on the system’s positioning accuracy and stability.

### 5.4. Error Comparison with an Existing Positioning Method

In Section 5.4, we compare the performance of the proposed method with an existing method introduced in [32].

To demonstrate the rationality of the parameter settings in the comparative experiment, we offer the following explanations: The UWB ranging error in [32] was set to 2 m, and the IMU used was the 9-DOF MPU-9150, with assumed displacement errors of 0.2 m and orientation errors of 0.1 rad. The rationality of the parameters has been previously confirmed in [32]. Therefore, we adopt the ranging error of 2 m from [32]. In addition, the IMU data used in this paper is from MPU-6050, whose data parameters are essentially the same as those of MPU-9150. Based on these considerations, we conclude that our parameter settings are appropriate for the purposes of our simulation.

**Figure 8:**
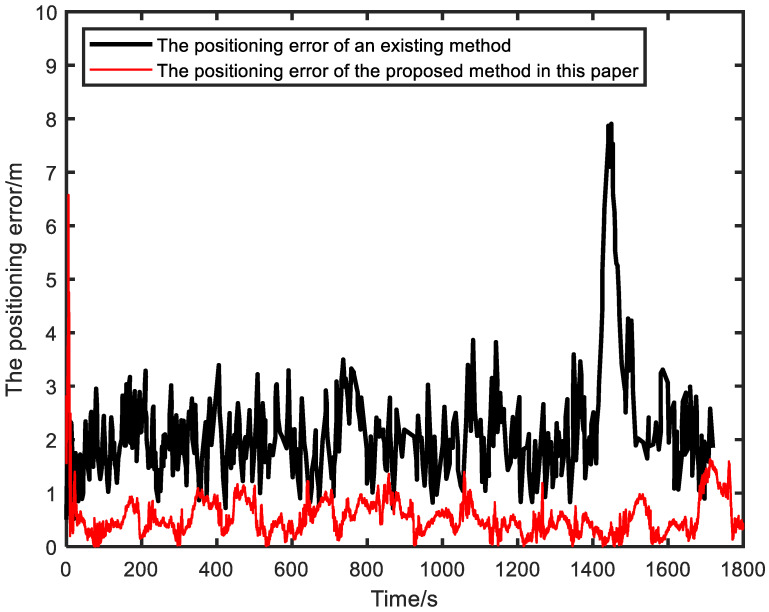
A comparison of the positioning errors between the method proposed in this paper and that in [32] is presented.

The experimental results of the two methods are shown in Figure 8. It can be observed that the average positioning error of the proposed method in this paper is approximately 0.55 m, while that of the method in [32] is about 2.14 m, which is approximately 3.89 times the error of the proposed method. In contrast, the relative positioning method proposed in this paper, which fuses ranging and IMU information, achieves better positioning accuracy.

At the initial startup phase of the positioning system, EKF may have uncertainty about the initial state, that is, the initial estimate of the system state may not be accurate enough, which may lead to large errors. As time goes on, since the system has started to operate, through measurement updates and filtering, the state estimate of the system will gradually become more accurate, and the error will gradually decrease until it stabilizes.

## 6. Conclusions

In this paper, we have presented a novel cooperative relative positioning method for MAVs in GNSS-denied scenarios. Within the framework of the system model, we have employed the EKF algorithm to fuse ranging and IMU information, addressing the problem of IMU error drift and enabling high-precision and robust MAV positioning. In addition, our proposed method has the following several distinctive features: it eliminates the requirement for precise reference anchors, expands the application scope of the system, and solves the problem of limited number of nodes in MAV formation. Furthermore, we have theoretically derived the system observability conditions and provided specific expressions that guarantee the system positioning accuracy when the system satisfies the observability conditions. We have verified the correctness of the observability theoretical analysis through simulations and investigated the influence of different ranging errors on the positioning accuracy and stability. Finally, we have demonstrated the superiority of the proposed method through simulation comparisons with an existing method.

We have verified the proposed method through simulation experiments. To ensure that the experiments are as close to reality as possible, the system parameters considered in this paper are taken from the official datasheet of the sensor and previous research [32,38,39,40]. However, there is still a certain gap between simulation and reality, mainly in the following aspects:

1. The presence of obstacles, wind conditions, and other environmental factors may affect the performance of MAVs in practical applications.
2. Simulated noise cannot fully reflect the noise experienced by MAVs in actual applications.
3. There may be a certain difference in air pressure at the same altitude but different horizontal positions due to the influence of factors such as atmospheric pressure, temperature, and humidity, which may result in some spatial variations. In addition, the scale of pressure gradient varies across different regions. As a result, the barometric altimeter readings may be affected and may introduce some measurement bias.
4. In practical applications, the airflow generated by the movement of MAVs may have an impact on the flight stability and control of MAVs.

These factors are difficult to model accurately in simulation experiments. Therefore, actual verification will direct our research in the future.

## Figures and Tables

**Table 1:** Descriptions of parameters or symbols in Section 2.

Parameters or Symbols	Descriptions
*n*	Earth-fixed North-East-Down (NED) coordinate system
*h*	Body-fixed horizontal coordinate system
*b*	Body-fixed coordinate system
φ	Yaw angle
θ	Pitch angle
γ	Roll angle
${\left\|\cdot \right\|}_{2}$	2-norm
$p_{i}^{n}\in {\mathbb{R}}^{3}$	Position vector of MAV *i* in *n*
$p_{ik}^{h_{i}}\in {\mathbb{R}}^{3}$	$\mathrm{Projection}\;\mathrm{of}\;\mathrm{the}\;\mathrm{relative}\;\mathrm{position}\;\mathrm{of}\;\mathrm{MAV}\;k\;\mathrm{with}\;\mathrm{respect}\;\mathrm{to}\;\mathrm{MAV}\;i\;\mathrm{in}\;h_{i}$
$C_{n}^{h_{i}}\in {\mathbb{R}}^{3\times 3}$	$\mathrm{Coordinate}\;\mathrm{transformation}\;\mathrm{matrix}\;\mathrm{from}\;n\;\mathrm{to}\;h_{i}$

**Table 2:** Descriptions of parameters or symbols in Section 3.

Parameters or Symbols	Descriptions
$x\in {\mathbb{R}}^{7}$	State vector
$u\in {\mathbb{R}}^{6}$	Input vector
$y\in {\mathbb{R}}^{5}$	Output vector
$\dot{x}=f\left(\cdot \right)$	State differential vector function
$h\left(\cdot \right)$	Observation function
$p\in {\mathbb{R}}^{2}$	$\mathrm{Projection}\;\mathrm{of}\;\mathrm{the}\;\mathrm{relative}\;\mathrm{position}\;\mathrm{of}\;\mathrm{MAV}\;2\;\mathrm{with}\;\mathrm{respect}\;\mathrm{to}\;\mathrm{MAV}\;1\;\mathrm{in}\;h_{1}$
$R\in {\mathbb{R}}^{2\times 2}$	Two-dimensional case of $C_{n}^{h_{i}}$
Δφ	Yaw angle difference between MAV 1 and MAV 2
$\Delta \dot{\varphi }$	Yaw rate difference between MAV 1 and MAV 2
$r_{i}$	Yaw rate of MAV *i*
$v_{i}\in {\mathbb{R}}^{2}$	$\mathrm{Projection}\;\mathrm{of}\;\mathrm{velocity}\;\mathrm{of}\;\mathrm{MAV}\;i\;\mathrm{in}\;h_{i}$
$a_{i}\in {\mathbb{R}}^{2}$	$\mathrm{Projection}\;\mathrm{of}\;\mathrm{acceleration}\;\mathrm{in}\;h_{i}$
$\omega_{i}\in {\mathbb{R}}^{3}$	Gyroscope measurement
$s_{i}\in {\mathbb{R}}^{3}$	Accelerometer measurement
$r_{i}^{\times }\in {\mathbb{R}}^{2\times 2}$	Antisymmetric matrix of cross product
$v_{i}^{n}\in {\mathbb{R}}^{2}$	Velocity of MAV *i* in *n*

**Table 3:** Descriptions of parameters or symbols in Section 4.

Parameters or Symbols	Descriptions
$L_{f}^{i}h$	The *i*-th order Lie derivative of ***h*** with respect to ***f***
$J\left(\cdot \right)$	Jacobi matrix
***H***	Observability matrix
$E_{i}$	The *i*-th order identity matrix
∂	Partial derivative
$A\in {\mathbb{R}}^{3\times 3}$	Combination matrix
$\left|\cdot \right|$	Determinant

**Table 4:** Parameter settings.

Parameters/(Unit)	Values
Filtering step/(s)	0.1
Total simulation duration/(s)	1000
Constant gyroscope drift/(rad/s)	0.05 deg/3600
$\mathrm{Constant}\;\mathrm{accelerometer}\;\mathrm{bias}/(m/s^{2}$)	0.1 mg
Barometric altimeter error/(m)	1
Ranging error/(m)	2
Velocity error/(m/s)	0.1
Noise matrix	$diag\left(0,0,10^{-8},10^{-6},10^{-6},10^{-6},10^{-6}\right)$
Initial error	${\left[2,2,2\deg,0.1,0.1,0.1,0.1\right]}^{T}$

**Table 5:** Motion parameter settings.

Parameters/(Unit)	Values
Rotational angular velocity of *AB*/(rad/s)	0.03
Sinusoidal angular velocity of *O*/(rad/s)	0.03
Linear velocity of *O*/(m/s)	20
Initial angle of MAV *A*/(rad)	π/3
Initial angle of MAV *B*/(rad)	π

**Table 6:** Mean statistical data from 100 Monte Carlo simulation tests.

	Parameters	+3σ Boundary	Mean	−3σ Boundary
Trajectory 1	*x*	1.62 m	−0.04 m	−1.69 m
	*y*	2.07 m	0.02 m	−2.03 m
Trajectory 2	*x*	8.47 m	2.44 m	−2.59 m
	*y*	4.19 m	1.39 m	−3.41 m
$\frac{\mathrm{Trajectory}\;2}{\mathrm{Trajectory}\;1}$	*x*	5.23	/	1.53
	*y*	2.02	/	1.18

**Figure A1:**
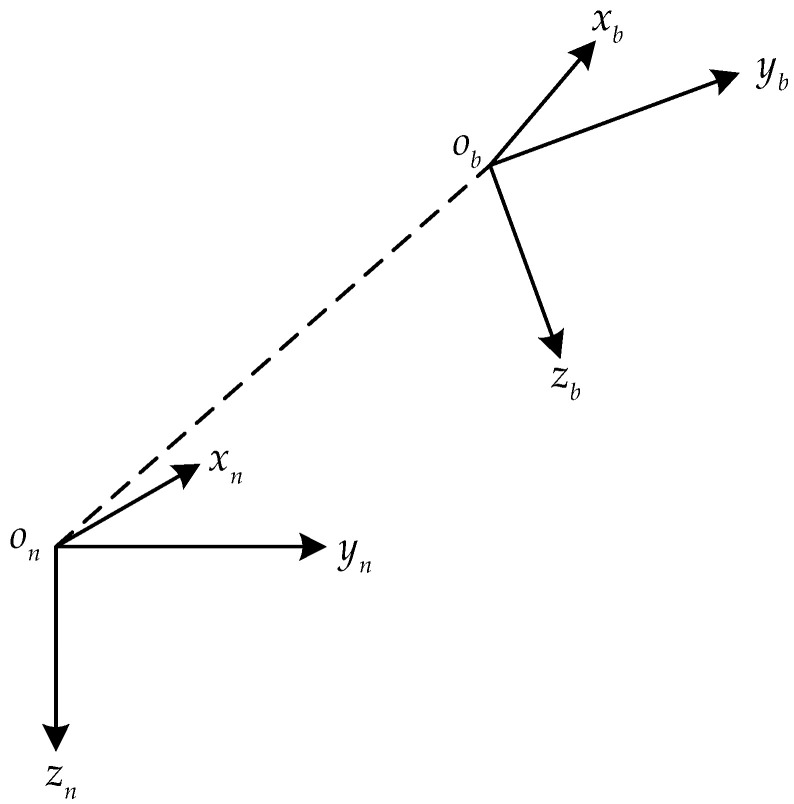
The relationship between the Earth-fixed North-East-Down (NED) coordinate system *n* and the body-fixed coordinate system *b*.

**Figure A2:**
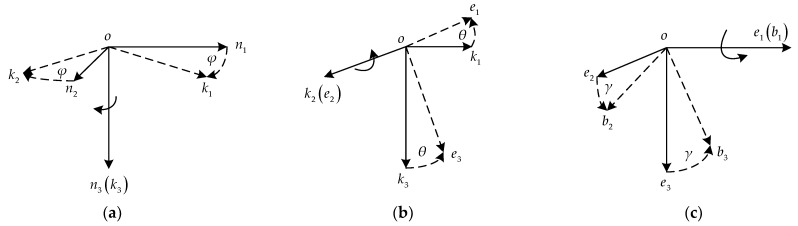
Define Euler angles by the 321-rotation sequence. (**a**) Yaw angle φ; (**b**) Pitch angle θ; (**c**) Roll angle γ.

## Data Availability

The data presented in this study are available on request from the corresponding author. The data are not publicly available due to the regulations of our laboratory.

## Appendix A

### Appendix A.1. Proof of Equation (5)

The relationship between the Earth-fixed North-East-Down coordinate system *n* and the typical body-fixed coordinate system *b* is shown in Figure A1. The following unit vectors are defined:

(A1) $$n_{1}≜{\left[\begin{array}{ccc} 1 & 0 & 0 \end{array}\right]}^{T},\;n_{2}≜{\left[\begin{array}{ccc} 0 & 1 & 0 \end{array}\right]}^{T},\;n_{3}≜{\left[\begin{array}{ccc} 0 & 0 & 1 \end{array}\right]}^{T}$$

The unit vectors along $o_{n}x_{n}$, $o_{n}y_{n}$, and $o_{n}z_{n}$ axes in *n* are represented by $n_{1}$, $n_{2}$, and $n_{3}$, respectively. The following relationships are satisfied by the unit vectors along $o_{b}x_{b}$, $o_{b}y_{b}$, and $o_{b}z_{b}$ axes in *b*:

(A2)
b1b=n1, b2b=n2, b3b=n3

Furthermore, the unit vectors along $o_{b}x_{b}$, $o_{b}y_{b}$, and $o_{b}z_{b}$ axes in *n* are represented by b1n, b2n, and b3n, respectively.

The schematic diagram of the rotational relationship from the Earth-fixed North-East-Down coordinate system *n* to the body-fixed coordinate system *b* is shown in Figure A2. Assuming $on_{1}n_{2}n_{3}$ is a right-handed Cartesian coordinate system, the following three rotations are performed: First, the $on_{1}n_{2}n_{3}$ system is rotated about the positive $on_{3}$-axis by an angle φ, resulting in the $ok_{1}k_{2}k_{3}$ system, which shares the same oz axis as the $on_{1}n_{2}n_{3}$ system. Next, the $ok_{1}k_{2}k_{3}$ system is rotated about the positive $ok_{2}$-axis by an angle θ, resulting in the $oe_{1}e_{2}e_{3}$ system, which shares the same oy axis as the $ok_{1}k_{2}k_{3}$ system. Finally, the $oe_{1}e_{2}e_{3}$ system is rotated about the positive $oe_{1}$-axis by an angle γ, resulting in the $ob_{1}b_{2}b_{3}$ system, which shares the same ox axis as the $oe_{1}e_{2}e_{3}$ system. The rotation sequence and the sign of each rotation angle can be denoted as ‘(+3)(+2)(+1)’, or simply ‘321’ by omitting the plus sign, where the numbers 1, 2, and 3 represent the rotations about ox, oy, and oz axes, respectively, and the plus sign in the parentheses indicates the positive direction of rotation according to the right-hand rule.

Assuming that the body angular velocity is ωb=ωxbωybωzbT, the relationship between the attitude change rate and the body angular velocity is given by the following equation [41]:

(A3) ωb=φ˙·k3b+θ˙·e2b+γ˙·b1b

where,

(A4) b1b=100T

(A5)
e2b=Reb⋅e2e=Reb⋅n2=Rxγ⋅n2=1000cosγsinγ0−sinγcosγ010=0cosγ−sinγT

(A6)
k3b=Rkb·k2k=Rkb⋅n3=RxγRyθ⋅n3=1000cosγsinγ0−sinγcosγcosθ0−sinθ010sinθ0cosθ001=−sinθsinγcosθcosγcosθT

According to Equations (A3)–(A6), the following equation is obtained:

(A7)
$$\left[\begin{array}{c} \omega_{xb}\\ \omega_{yb}\\ \omega_{zb} \end{array}\right]=\left[\begin{array}{ccc} 1 & 0 & -\sin\theta \\ 0 & \cos\gamma & \sin\gamma \cos\theta \\ 0 & -\sin\gamma & \cos\gamma \cos\theta \end{array}\right]\left[\begin{array}{c} \dot{\gamma }\\ \dot{\theta }\\ \dot{\varphi } \end{array}\right]$$

Then we can obtain the following expression by applying the inverse transformation to Equation (A7):

(A8) $$\begin{array}{ll} \left[\begin{array}{c} \dot{\gamma }\\ \dot{\theta }\\ \dot{\varphi } \end{array}\right] & ={\left[\begin{array}{ccc} 1 & 0 & -\sin\theta \\ 0 & \cos\gamma & \sin\gamma \cos\theta \\ 0 & -\sin\gamma & \cos\gamma \cos\theta \end{array}\right]}^{-1}\left[\begin{array}{c} \omega_{xb}\\ \omega_{yb}\\ \omega_{zb} \end{array}\right]\\ & =\left[\begin{array}{ccc} 1 & \sin\gamma \tan\theta & \cos\gamma \tan\theta \\ 0 & \cos\gamma & -\sin\gamma \\ 0 & \frac{\sin\gamma }{\cos\theta } & \frac{\cos\gamma }{\cos\theta } \end{array}\right]\left[\begin{array}{c} \omega_{xb}\\ \omega_{yb}\\ \omega_{zb} \end{array}\right] \end{array}$$

where,

(A9) $$\dot{\varphi }=\frac{\sin\gamma }{\cos\theta }\omega_{yb}+\frac{\cos\gamma }{\cos\theta }\omega_{zb}$$

Since the yaw angles in both coordinate systems (*h* and *b*) are identical, the yaw rate in the horizontal body-fixed coordinate system *h* is the same as that given by Equation (A9). Therefore, Equation (5) is confirmed.

### Appendix A.2. Proof of Equation (6)

According to the relationship between the direction cosine matrix and the equivalent rotation vector, the transformation matrix from *b* to *n* in Figure A2 can be obtained as follows:

(A10) $$\begin{array}{l} C_{b}^{n}=\left[\begin{array}{ccc} \cos\varphi & -\sin\varphi & 0\\ \sin\varphi & \cos\varphi & 0\\ 0 & 0 & 1 \end{array}\right]\left[\begin{array}{ccc} \cos\theta & 0 & \sin\theta \\ 0 & 1 & 0\\ -\sin\theta & 0 & \cos\theta \end{array}\right]\left[\begin{array}{ccc} 1 & 0 & 0\\ 0 & \cos\gamma & -\sin\gamma \\ 0 & \sin\gamma & \cos\gamma \end{array}\right]\\ =\left[\begin{array}{ccc} \cos\varphi \cos\theta & -\sin\varphi \cos\gamma +\cos\varphi \sin\theta \sin\gamma & \sin\varphi \sin\gamma +\cos\varphi \sin\theta \cos\gamma \\ \sin\varphi \cos\theta & \cos\varphi \cos\gamma +\sin\varphi \sin\theta \sin\gamma & -\cos\varphi \sin\gamma +\sin\varphi \sin\theta \cos\gamma \\ -\sin\theta & \cos\theta \sin\gamma & \cos\theta \cos\gamma \end{array}\right] \end{array}$$

In this paper, we use the fixed-body horizontal coordinate system *h*, whose yaw angle is the same as that of the typical fixed-body coordinate system *b*, and *x*-*y* plane is parallel to that of the NED coordinate system *n*. Therefore, the transformation matrix from *b* to *h* is equivalent to $C_{b}^{n}$ with φ=0, which is given by:

(A11)
$$C_{b}^{h}=\left[\begin{array}{ccc} \cos\theta & \sin\theta \sin\gamma & \sin\theta \cos\gamma \\ 0 & \cos\gamma & -\sin\gamma \\ -\sin\theta & \cos\theta \sin\gamma & \cos\theta \cos\gamma \end{array}\right]$$

Since this paper considers the two-dimensional case, according to Equation (A11), the transformation matrix of Equation (6) can be obtained.

## References

[1] Suzuki T., Matsuo K., Amano Y. (2020). Rotating GNSS Antennas: Simultaneous LOS and NLOS Multipath Mitigation. GPS Solut..

[2] Hermosilla T., Palomar-Vázquez J., Balaguer-Beser Á., Balsa-Barreiro J., Ruiz L.A. (2014). Using street based metrics to characterize urban typologies. Comput. Environ. Urban Syst..

[3] Nicola M., Falco G., Morales Ferre R., Lohan E.-S., de la Fuente A., Falletti E. (2020). Collaborative Solutions for Interference Management in GNSS-Based Aircraft Navigation. Sensors.

[4] José B.B. (2014). Aplicación de Sistemas GNSS y SIG a Infraestructuras de Transporte: Estudio Sobre la Conducción Naturalista. Ph.D. Thesis.

[5] Wang S., Dong X., Liu G., Gao M., Xiao G., Zhao W., Lv D. (2022). GNSS RTK/UWB/DBA Fusion Positioning Method and Its Performance Evaluation. Remote Sens..

[6] He S., Hu S., Guo Q., Jiang W. (2022). Relative Positioning Method for UAVs Based on Multi-Source Information Fusion. Math. Probl. Eng..

[7] Zhang J., Ren M., Wang P., Meng J., Mu Y. (2020). Indoor Localization Based on VIO System and Three-Dimensional Map Matching. Sensors.

[8] Zhu F., Shen Y., Wang Y., Jia J., Zhang X. (2021). Fusing GNSS/INS/Vision With A Priori Feature Map for High-Precision and Continuous Navigation. IEEE Sens. J..

[9] Naus K., Waz M. (2016). Precision in Determining Ship Position using the Method of Comparing an Omnidirectional Map to a Visual Shoreline Image. J. Navig..

[10] Krüger R., Simeonov G., Beck F., Ertl T. (2018). Visual Interactive Map Matching. IEEE Trans. Vis. Comput. Graph..

[11] Yoo D.-H., Shan G., Roh B.-H. (2022). A Vision-based Indoor Positioning Systems utilizing Computer Aided Design Drawing. Proceedings of the 28th Annual International Conference on Mobile Computing and Networking (ACM MobiCom ’22).

[12] Xiong J., Cheong J.W., Ding Y., Xiong Z., Dempster A.G. (2022). Efficient Distributed Particle Filter for Robust Range-Only SLAM. IEEE Internet Things J..

[13] Wang Y., Wang X. (2022). Research on SLAM Road Sign Observation Based on Particle Filter. Comput. Intell. Neurosci..

[14] Lu S., Zhi Y., Zhang S., He R., Bao Z. (2021). Semi-Direct Monocular SLAM With Three Levels of Parallel Optimizations. IEEE Access.

[15] Munguia R., Trujillo J.-C., Guerra E., Grau A. (2022). A Hybrid Visual-Based SLAM Architecture: Local Filter-Based SLAM with KeyFrame-Based Global Mapping. Sensors.

[16] Balsa-Barreiro J., Fritsch D. (2018). Generation of visually aesthetic and detailed 3D models of historical cities by using laser scanning and digital photogrammetry. Digit. Appl. Archaeol. Cult. Herit..

[17] Salarian M., Iliev N., Çetin A.E., Ansari R. (2018). Improved Image-Based Localization Using SFM and Modified Coordinate System Transfer. IEEE Trans. Multimed..

[18] Xing H., Zhao Y., Zhang Y., Chen Y. (2020). 3d trajectory planning of positioning error correction based on pso-a* algorithm. Comput. Mater. Contin..

[19] Chen D., Neusypin K., Selezneva M., Mu Z. (2019). New Algorithms for Autonomous Inertial Navigation Systems Correction with Precession Angle Sensors in Aircrafts. Sensors.

[20] Cho S.Y., Lee J.H., Park C.G. (2022). A Zero-Velocity Detection Algorithm Robust to Various Gait Types for Pedestrian Inertial Navigation. IEEE Sens. J..

[21] Wang Q., Liu K., Sun Z., Cai M., Cheng M. (2019). Research on the Heading Calibration for Foot-Mounted Inertial Pedestrian-Positioning System Based on Accelerometer Attitude. Electronics.

[22] Chen H., Taha T.M., Chodavarapu V.P. (2022). Towards Improved Inertial Navigation by Reducing Errors Using Deep Learning Methodology. Appl. Sci..

[23] Maheepala M., Joordens M.A., Kouzani A.Z. (2022). A Low-Power Connected 3-D Indoor Positioning Device. IEEE Internet Things J..

[24] Lin S.-H., Chang Chien H.-H., Wang W.-W., Lin K.-H., Li G.-J. (2022). An Efficient IAKF Approach for Indoor Positioning Drift Correction. Sensors.

[25] Park J., Kim Y.-J., Lee B.K. (2020). Passive Radio-Frequency Identification Tag-Based Indoor Localization in Multi-Stacking Racks for Warehousing. Appl. Sci..

[26] Peng Z., Cheng S., Li X., Li K., Cai M., You L. Dynamic Visual SLAM Integrated with IMU for Unmanned Scenarios. Proceedings of the 2022 IEEE 25th International Conference on Intelligent Transportation Systems (ITSC).

[27] Yu F., Yu H., Wei Y. Research on Robot Positioning Technology Based on Multi Sensor. Proceedings of the 2019 International Conference on Computer Network, Electronic and Automation (ICCNEA).

[28] Olsson F., Rantakokko J., Nygårds J. Cooperative localization using a foot-mounted inertial navigation system and ultrawideband ranging. Proceedings of the 2014 International Conference on Indoor Positioning and Indoor Navigation (IPIN).

[29] Liu R., Yuen C., Do T.-N., Jiao D., Liu X., Tan U.-X. Cooperative relative positioning of mobile users by fusing IMU inertial and UWB ranging information. Proceedings of the 2017 IEEE International Conference on Robotics and Automation (ICRA).

[30] Long K., Shen C., Tian C., Zhang K., Bhatti U.A., Kong D.F.N., Feng S., Cheng H. (2021). Single UWB Anchor Aided PDR Heading and Step Length Correcting Indoor Localization System. IEEE Access.

[31] Zhao X., Yan G., Chang T., Liang C.H., Wang Z., Fu H. Antenna design for ultra-wideband through wall radar. Proceedings of the 2017 3rd IEEE International Conference on Computer and Communications (ICCC).

[32] Xu H., Zhu Y., Wang G. On the anti-multipath performance of UWB signals in indoor environments. Proceedings of the ICMMT 4th International Conference on Microwave and Millimeter Wave Technology.

[33] Lou X., Zhao Y. High-Accuracy Positioning Algorithm Based on UWB. Proceedings of the 2019 International Conference on Artificial Intelligence and Advanced Manufacturing (AIAM).

[34] Yuan K., Wang H., Zhang H. Robot Position Realization Based on Multi-sensor Information Fusion Algorithm. Proceedings of the 2011 Fourth International Symposium on Computational Intelligence and Design.

[35] Zheng W., Wang J., Wang Z. Multi-sensor fusion based real-time hovering for a quadrotor without GPS in assigned position. Proceedings of the 2016 Chinese Control and Decision Conference (CCDC).

[36] Zhou X.S., Roumeliotis S.I. (2008). Robot-to-Robot Relative Pose Estimation from Range Measurements. IEEE Trans. Robot..

[37] Velimir J. (1970). Abstract control systems: Controllability and observability. SIAM J. Control.

[38] Cismas A., Ioana M., Vlad C., Casu G. Crash Detection Using IMU Sensors. Proceedings of the 2017 21st International Conference on Control Systems and Computer Science (CSCS).

[39] Chen M., Luo W., Xu Z., Zhang X., Xie B., Wang G., Han M. (2019). An ultrahigh resolution pressure sensor based on percolative metal nanoparticle arrays. Nat. Commun..

[40] Silva B., Hancke G.P. (2020). Ranging Error Mitigation for Through-the-Wall Non-Line-of-Sight Conditions. IEEE Trans. Ind. Inform..

[41] Ducard G.J.J. (2009). Fault-Tolerant Flight Control and Guidance Systems: Practical Methods for Small Unmanned Aerial Vehicles.

